# Safe surgery in the elderly: A review of outcomes following robotic proctectomy from the Nationwide Inpatient Sample in a cross-sectional study

**DOI:** 10.1016/j.amsu.2019.06.004

**Published:** 2019-06-20

**Authors:** Carly R. Richards, Scott R. Steele, Michael B. Lustik, Suzanne M. Gillern, Robert B. Lim, Justin T. Brady, Ali R. Althans, Andrew T. Schlussel

**Affiliations:** a Department of Surgery, Tripler Army Medical Center, Honolulu, HI, United States; b Department of Colon & Rectal Surgery, Cleveland Clinic Foundation, Cleveland, OH, United States; c Department of Clinical Investigations, Tripler Army Medical Center, Honolulu, HI, United States; d Department of Surgery, University Hospitals Case Medical Center, Cleveland, OH, United States; e Department of Surgery, Madigan Army Medical Center, Tacoma, WA, United States

**Keywords:** Robotic, Proctectomy, Elderly, National in-patient sample(NIS)

## Abstract

**Background:**

As our nation's population ages, operating on older and sicker patients occurs more frequently. Robotic operations have been thought to bridge the gap between a laparoscopic and an open approach, especially in more complex cases like proctectomy.

**Methods:**

Our objective was to evaluate the use and outcomes of robotic proctectomy compared to open and laparoscopic approaches for rectal cancer in the elderly. A retrospective cross-sectional cohort study utilizing the Nationwide Inpatient Sample (NIS; 2006–2013) was performed. All cases were restricted to age 70 years old or greater.

**Results:**

We identified 6740 admissions for rectal cancer including: 5879 open, 666 laparoscopic, and 195 robotic procedures. The median age was 77 years old. The incidence of a robotic proctectomy increased by 39%, while the open approach declined by 6% over the time period studied. Median (interquartile range) length of stay was shorter for robotic procedures at 4.3 (3–7) days, compared to laparoscopic 5.8 (4–8) and open at 6.7 (5–10) days (p < 0.01), while median total hospital charges were greater in the robotic group compared to laparoscopic and open cases ($64,743 vs. $55,813 vs. $50,355, respectively, p < 0.01). There was no significant difference in the risk of total complications between the different approaches following multivariate analysis.

**Conclusion:**

Robotic proctectomy was associated with a shorter LOS, and this may act as a surrogate marker for an overall improvement in adverse events. These results demonstrate that a robotic approach is a safe and feasible option, and should not be discounted solely based on age or comorbidities.

## Highlights

• Robotic proctectomy in the elderly appears to be safe practice.
• Open proctectomy is still by far the most common approach.
• The use of robotics for proctectomy is still quite low but growing substantially.

## 1 Introduction

As the nation's population ages, the volume of surgical interventions on older patients has continued to grow. The United States census data has shown that from 1980 to 2010, the age of citizens 65–89 years old has doubled, and those greater than 90 years old has almost tripled [1]. This increase in life expectancy presents the challenge of continuing to deliver safe and effective health care to not only the elderly, but those with a significant number of medical comorbidities [2]. These patients often require complex medical and surgical decision making to achieve the most successful outcomes and to maintain a functional quality of life.

Open colorectal surgery in the elderly has been associated with increased morbidity and mortality when compared with younger patients [3,4]. With a decreased physiologic reserve and an increased number of comorbidities, the application of laparoscopy has become particularly important to this population. This technique is associated with a decreased length of stay (LOS), decreased morbidity rate, earlier return of bowel function, and equivalent oncologic outcomes as compared to open surgery for patients greater than 70 years old [[5], [6], [7], [8]]. Laparoscopic rectal surgery, however, has a steeper learning curve as compared to a laparoscopic colectomy due to the complex anatomical nature of the pelvis, and relatively poor ergonomics of laparoscopic instruments [9]. Furthermore, laparoscopic proctectomy is associated with a greater rate of conversion to an open procedure, which may subsequently lead to long-term complications. In addition, studies have demonstrated an increased risk of bladder and sexual dysfunction when compared to an open approach [10,11].

The da Vinci surgical system (da Vinci Surgical System; Intuitive Surgical Inc.) was approved by the US Food and Drug Administration in 2000 as a robotic surgical device. This operating system was designed in an effort to help minimize the learning curve experienced by many surgeons when transitioning to a minimally invasive approach. This platform was created to improve visualization and stability, increase the freedom of movement, and maximize dexterity [12]. Because these features eliminate many of the technical limitations of laparoscopy, robotic operations have been proposed to bridge the gap between open and laparoscopic surgery, thus facilitating the dispersion of minimally invasive techniques to a broader population. Some data on robotic proctectomy has shown the robotic approach to decrease LOS, postoperative complications, conversion rates, and circumferential margin involvement when compared to traditional laparoscopy [[13], [14], [15], [16]]. However data from the highly anticipated ROLARR (Robotic versus Laparoscopic Resection for Rectal Cancer) trial showed no benefit to robotic surgery in reducing the conversion to open in curative resection for rectal adenocarcinoma. It did show that laparoscopic and robotic assisted laparoscopic were equivalent as far as outcomes studied [17]. Although the use of a minimally invasive technique appears to be especially beneficial for the elderly population, there is a paucity of literature describing robotic proctectomy for this cohort [16]. Therefore, we aimed to evaluate both the trend in utilization and surgical outcomes following robotic proctectomy for rectal cancer among patients ages 70 years or greater in the United States.

## 2 Materials and methods

The Human Use Committee approved the study protocol. This work has been reported in line with the STROCSS criteria; it has been registered with ClinicalTrials.gov, identification number NCT03765411 [18]. Investigators adhered to the policies for protection of human subjects as prescribed in 45 Code of Federal Regulation 46. The Nationwide Inpatient Sample (NIS) database is the largest all-payer inpatient care database in the United States including persons covered by Medicare, Medicaid, private insurance and the uninsured, with data from over 8 million hospital admissions each year. This dataset allows for accurate national estimates from approximately 20% of all discharges nationwide. It includes admission and discharge diagnoses, procedures performed and complications and outcome data during the hospitalization [19]. Data was collected from the NIS, a part of the Health Care Utilization Project (HCUP), Association for Healthcare Research and Quality (AHRQ) from 2006 to 2013.

### 2.1 Definition of variables

Patients 70 years of age or older were identified by primary *ICD-*9-CM procedure codes for proctectomy (48.62, 48.63, 48.64, 48.69). We excluded all emergent admissions (NIS ELECTIVE ≠ 1) and those patients undergoing abdominoperineal resections (48). Patients were categorized by *ICD-*9-CM procedure codes for operative approach: robotic (48.62, 48.63, 48.64, 48.69 + 17.41, 17.42, or 17.49), laparoscopic (48.62, 48.63, 48.64, 48.69 + 54.21), and open (48.62, 48.63, 48.64, 48.69). Conversion from laparoscopic or robotic to open was documented. All cases were restricted to patients with an *ICD-*9-CM diagnosis code for rectal cancer (154.0, 154.1, 153.9, 198.8) (Table 1). Trends in utilization of each approach were determined, and in-hospital outcomes were compared between the minimally invasive cohorts and an open approach. All variables and outcomes with a sample size less than 10 were excluded per NIS guidelines.

**Table 1:** Procedure codes and operative group designator Code(s).

Procedure	ICD-9-CM Procedure Code
Anterior resection of the rectum with synchronous colostomy	48.62
Anterior resection of the rectum, other	48.63
Posterior resection of the rectum	48.64
Resection of the rectum, other	48.69

Operative Approach	Designator ICD-9-CM Procedure Codes
Open	No additional codes
Laparoscopic	54.21
Robotic	17.42 (Laparoscopic robotic assisted)
	17.49 (Other/unspecified robotic assisted)

### 2.2 Demographics

Demographic data collected by NIS includes: age, gender, race, disease stage, household income zip code quartile, obesity (yes or no), hospital bed side (small, medium, large), days from admission to procedure, comorbidities, disposition (home, short-term hospital, home health care, against medical advice, died in hospital, discharged alive destination unknown), insurance status, geographic region, and teaching status/location of the hospital (urban versus rural).

### 2.3 Comorbidities

Comorbidities identified included: arthritis, congestive heart failure, chronic obstructive pulmonary disease, coagulopathy, diabetes with or without chronic complications, hypertension, liver disease, metastatic cancer, morbid obesity, non-morbid obesity, peripheral vascular disease, pulmonary circulation disease, renal failure, solid tumor without metastases, and weight loss. In addition, comorbidities were assessed using the Elixhauser Comorbidity index (ECI). The ECI is based on the Charlson comorbidity index, which predicts the one year mortality for patients with certain conditions [20]. Unlike the Charlson Comorbidity index, which includes 17 comorbidities, the ECI includes 30 variables. The Elixhauser Index is a combination of these 30 comorbidities, identified via *ICD-*9-CM codes, but excludes the diagnosis related group (DRG) [21].

The All Patient Refined Disease Related Group (APR-DRG) (3 M™ Health Information Systems) is a measure to compare a patient's risk of mortality (ROM) and severity of illness (SOI). DRG correlates with the patient's main diagnosis but is categorized into specific diagnoses that can be compared across cohorts. The APR-DRG was developed from the Medicare/Medicaid DRG prospective payment system to provide a risk-adjustment tool, garnered from clinical models based on historical data. Categories utilized to determine this score include age, type of surgical procedure, comorbidities, and the principle diagnosis. A SOI and ROM score is assigned to each surgical procedure and is indicated as minor [1], moderate [2], major [3], or extreme [4]. The software is proprietary, however, the methodology used to determine this score has been validated and used as a mean of risk-adjustment in previous studies [[22], [23], [24], [25]].

### 2.4 Age

Age was analyzed as a continuous variable for all comparisons. Cases were separated into five groups: 70–74, 75–79, 80–84, 85–89, and 90 years of age and older.

### 2.5 Race

Race was defined at Caucasian, African American, Hispanic, other, and unknown. Patients for whom data was unavailable were classified into unknown.

### 2.6 Insurance status

Patients were categorized by insurance status: Medicare, Medicaid, or private insurance. Patients who did not have any form of insurance (i.e. self-pay) were grouped together as “other.”

### 2.7 Disease stage

Disease stage was divided into localized, locally advanced, regionally nodal, and metastatic. These categories were further divided by HCUP and AHRQ criteria based on size and spread of disease.

### 2.8 In-hospital complications

Complications were analyzed by system and included: mechanical wound, infections, urinary, pulmonary, gastrointestinal, cardiovascular, systemic, surgical, and any complication. Specific complications were also evaluated and included: acute kidney injury, cardiac arrest, deep venous thrombosis, myocardial infarction, pneumonia, pulmonary embolism, sepsis/septic shock, stroke, surgical sight infection, postoperative intubation, and urinary tract infection.

### 2.9 Ostomy creation

Ostomy creation was evaluated. Patients who underwent ostomy creation were separated into ileostomy and colostomy.

### 2.10 In-hospital mortality

In-hospital mortality was evaluated as a secondary outcome measure. Data for patients collected by NIS is only available until discharge. Therefore, any death within 30 days of a procedure, but after discharge, was not available.

### 2.11 Length of stay

Length of stay was measured in days. The median and interquartile range (IQR) were compared between groups.

### 2.12 Costs and charges

Hospital charges refer to the total amount billed by the hospital. Hospital cost refers to the amount paid by the insurance provider. Medians (IQR) were compared between groups.

### 2.13 Statistical analysis

All statistical analyses were performed using SAS software version 9.2 (SAS Institute, Cary, NC). Unless otherwise noted, results are presented as unadjusted frequencies and a weighted percent because the NIS database is a 20% sample of yearly inpatient admission. To account for the complex sampling design of NIS, SAS SURVEYFREQ and SURVEYLOGISTIC statistical procedures with subdomain analysis were used to analyze categorical dependent variables. SURVEYMEANS and SURVEYREG were used for continuous dependent variables, per guidance of Healthcare Cost and Utilization Project NIS tutorials. The percent of missing data was less than 1% for all variables except race (15%), disease stage (33%), days from admission to procedure (10%) and household income (2%). Missing data for race and income were classified as “unknown” for analysis.

Chi-square tests and t-tests were used to compare demographics and outcomes between the robotic group and the laparoscopic and open groups. Multivariable logistic regression was used for adjusted analyses of complications. Because patients were not randomly assigned to surgical procedures, a propensity model was developed to address potential selection bias. This model was used to predict the estimated propensity for undergoing a robotic procedure versus a lap or open procedure based on demographic data, diagnosis, and hospital characteristics for the intended operation. The estimated propensity score from this model was added into the adjusted models as a predictor for the outcome variables. Total hospital charges and LOS were also compared. A stepwise approach based on a significance level of p < 0.05 was adopted for including variables in a final adjusted model. Age, sex, and the ECI were forced into the adjusted analyses regardless of significance as these were felt to be clinically significant. Adjusted odds ratios (ORs) are reported with 95% confidence intervals (CIs) or Interquartile Ranges (IQR). Variables with multiple categories are reported with OR referenced to the first category. A significance level p < 0.05 was used for all analyses.

## 3 Results

### 3.1 Demographics

**Fig. 1:**
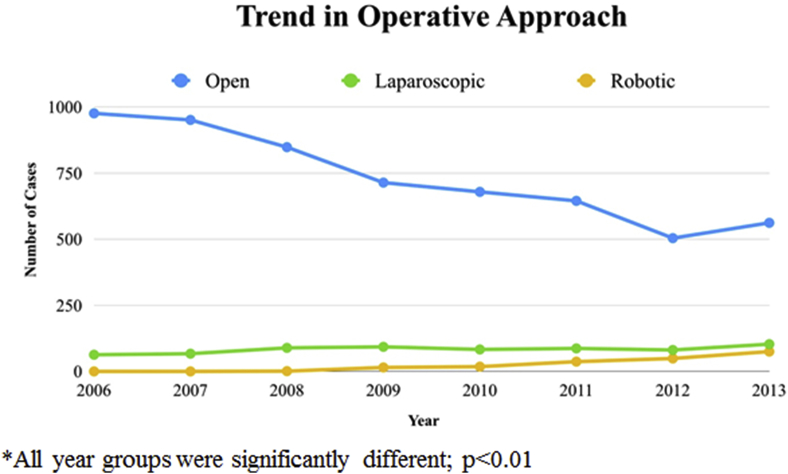
The above figure shows the annual number of proctectomy cases (procedure codes defined in Table 1), separated by operative approach.

We identified 6740 admissions for patients receiving a proctectomy for rectal cancer. The operative approach included 5879 open, 666 laparoscopic, and 195 robotic procedures. The median age was 77 years old (IQR 73–82), 54% of cases were men, and 3% of patients were greater than 90 years old. Utilization of both laparoscopic (1% median change in weighted percent over 7 years, IQR 0–3%) and robotic (8.5% median change in weighted percent over 7 years, IQR 8–11%) approaches increased significantly when compared to an open procedure (−1% median change in weighted percent over 7 years, IQR -2 - 0%) over the time course of this study (p < 0.01). The overall rate of open proctectomy decreased from 1039 to 740 cases, a reduction in weighted percent from 15% to 11% (please see the methods section for further explanation of NIS weighted percent). The open approach declined from 16% to 10%, and the laparoscopic approach increased from 9% to 16% over time. The utilization of the robotic platform increased from zero reported cases in 2006 to 75 in 2013 (Table 2; Fig. 1).

**Table 2:** Demographics.

	All (n = 6740) n (weighted %)	Open (n = 5879) n (weighted %)	Robotic (n = 195) n (weighted %)	Laparoscopic (n = 666) n (weighted %)	p-value	
					Open vs. Robotic	Lap vs. Robotic
Age						
70-74	2238 (33)	1920 (33)	87 (45)	231 (35)	**< 0.01**	0.109
75-79	2001 (30)	1753 (30)	52 (27)	196 (29)		
80-84	1507 (22)	1315 (22)	37 (19)	155 (23)		
85-89	775 (12)	688 (12)	*	72 (11)		
90+	219 (3)	203 (3)	*	12 (2)		
median (IQR)	77 (73–82)	77 (73–82)	75 (72–80)	77 (72–81)	**<0.01**	**0.01**
Gender						
Male	3634 (54)	3139 (54)	105 (54)	390 (59)	0.917	0.247
Female	3099 (46)	2733 (46)	90 (46)	276 (41)		
Race/ethnicity						
White	4654 (69)	4061 (69)	133 (68)	460 (69)	**0.02**	0.841
Black	314 (5)	275 (5)	*	30 (4)		
Hispanic	389 (6)	319 (5)	17 (9)	53 (8)		
Other	373 (5)	307 (5)	18 (9)	48 (7)		
Unknown	1010 (15)	917 (16)	18 (9)	75 (11)		
Region						
Northeast	1437 (22)	1259 (22)	37 (19)	141 (21)	0.884	0.524
Midwest	1517 (23)	1320 (23)	49 (25)	148 (22)		
South	2487 (37)	2194 (37)	74 (37)	219 (33)		
West	1299 (19)	1106 (18)	35 (18)	158 (23)		
Medicare						
No	552 (8)	461 (8)	22 (11)	69 (10)	0.093	0.682
Yes	6183 (92)	5414 (92)	173 (89)	596 (90)		
Location/teaching status						
Rural	10 (678)	11 (634)	*	42 (6)	**<0.01**	**<0.01**
Urban non-teaching	43 (2878)	43 (2536)	57 (30)	285 (43)		
Urban teaching	47 (3165)	46 (2697)	134 (69)	334 (51)		
Hospital bed size						
Small	11 (754)	11 (672)	19 (9)	63 (9)	0.827	0.919
Medium	25 (1635)	25 (1424)	45 (24)	166 (25)		
Large	65 (4332)	64 (3771)	129 (67)	432 (66)		
Disposition						
Routine	50 (3330)	50 (2864)	98 (52)	368 (56)	**<0.01**	**0.01**
Home Health Care	26 (1721)	26 (1488)	68 (36)	165 (25)		
Transfer	23 (1539)	24 (1397)	24 (13)	118 (18)		
Disease Stage						
Localized	68 (3096)	68 (2788)	30 (89)	278 (71)	0.063	0.152
Locally advanced	4 (172)	4 (155)	*	17 (4)		
Regionally nodal	15 (700)	16 (644)	*	52 (13)		
Metastatic	12 (562)	13 (517)	*	44 (11)		
Elixhauser						
mean (std)	6.6	6.7	5.2	5.9	**0.02**	0.31
median (IQR)	3.4 (−1.1–11.6)	3.8 (−1.1–11.6)	−0.1 (−1.3–11.3)	2.4 (−1.2–11.3)		

* indicates sample size less than 10. Bolded p values are statistically significant.

### 3.2 Comorbidities

The mean Elixhauser Comorbidity Index was significantly greater in the open vs. robotic cohort (6.7 CI 6.5–6.9 vs. 5.2 CI 4–6.3; p = 0.02), with no significant difference when comparing the robotic to laparoscopic groups. The APR-DRG risk of mortality and severity scores differed between all three groups: weighted mean open 53.75 and 55.75, weighted mean laparoscopic 50.25 and 53, and weighted mean robotic 44.5 and 46.75 respectively. The open group had the greatest risk of mortality when compared to the laparoscopic and robotic cohorts (Open CI 53–54, Laparoscopic CI 48–52, and Robotic CI 41–48; p < 0.01).

### 3.3 Outcomes

The rate of a complication following proctectomy was 22% (all approaches combined), with the majority of complications related to the gastrointestinal system (9.4%). A robotic approach was associated with a significantly reduced risk of total complications when compared to an open (13% vs. 23%; p < 0.01) and laparoscopic (21% vs. 13%; p = 0.01) approach. There was no significant difference in the risk of total complications following multivariate analysis (robotic versus open: OR 0.66 CI 0.4–1, p = 0.06; robotic versus laparoscopic: OR 0.66 CI 0.4–1, p = 0.09). The mortality rate was low (2.1%) with no significant difference among the groups (p = 0.9 for both comparisons). All remaining outcome measures evaluated had a sample size less than ten and therefore could not be statistically compared due to NIS database guidelines. Median (IQR) LOS was shorter in the robotic cohort at 4.3 (3–7) days, compared to the laparoscopic [5.8 (4–8)] and open [6.7 (5–10)] groups (p < 0.01).

### 3.4 Costs and charges

The median (IQR) total hospital charges were greater in the robotic group $64,743 ($44,731-$98,397) compared to laparoscopic $55,813 ($38,402-$82,069) and open groups $50,355 ($33,376-$81,231) (p < 0.01). The variation in cost between different surgical approaches was significantly less than the differences in charges between different surgical approaches. However, there was still a significant difference in cost noted between groups. Findings demonstrated the median (IQR) cost for a robotic procedure to be $18,889 ($15,179-$26,599), a laparoscopic procedure $21,270 ($12,491-$24,380), and an open procedure $16,134 ($11,712-$24,146) (p < 0.01) (Table 3).

**Table 3:** Comorbidities by Operative Approach (in weighted percentage). Bolded p values are statistically significant.

Comorbidity	All	Open	Robotic	Laparoscopic	p-value	
					Open vs. Robotic	Lap vs. Robotic
Arthritis	1.5	1.4	1.0	2.0	0.58	0.33
CHF	10.0	10.5	4.5	7.5	***0.01***	0.13
Chronic pulmonary disease	18.0	18.4	13.4	16.1	0.06	0.35
Coagulapathy	2.9	3.0	3.2	2.5	0.88	0.61
Diabetes with chronic complications	2.3	2.4	0.5	2.6	0.08	0.07
Hypertension	66.0	65.8	65.9	67.7	0.99	0.62
Liver disease	1.1	1.1	0.5	2.0	0.47	0.17
Metastatic cancer	25.9	26.4	15.3	24.8	***<0.01***	***0.01***
Obesity	6.6	6.3	6.2	9.1	0.97	0.21
Peripheral vascular disease	6.4	6.5	5.9	6.0	0.74	0.99
Pulmonary circulation disease	2.0	2.0	2.0	2.0	0.98	1.00
Renal failure	7.3	7.3	7.6	6.6	0.88	0.61
Solid tumor w/o metastasis	4.1	4.2	3.5	2.6	0.64	0.50
Weight loss	7.1	7.2	8.2	5.6	0.61	0.20
Diabetes (with or without complications)	24.0	24.3	22.0	22.1	0.46	0.98
COPD	13.8	14.3	10.6	10.6	0.14	0.99
Morbid obesity	1.7	1.7	1.6	2.2	0.90	0.62
Nonmorbid obesity	5.1	4.8	5.7	7.6	0.56	0.37

Elixhauser						
mean (std)	6.6	6.7	5.2	5.9	***0.02***	0.31
median (IQR)	3.4 (−1.1–11.6)	3.8 (−1.1–11.6)	−0.1 (−1.3–11.3)	2.4 (−1.2–11.3)		

## 4 Discussion

As technology progresses it is critical that surgeons continue to build their armamentarium of techniques in efforts to provide the most safe and effective care to all patients. These results have demonstrated an increased trend in the utilization of the robotic platform, with findings that are consistent with the current literature. Damle et al. identified a 17% increase in robotic assisted colorectal surgery (RACS) between 2011 and 2015 while Lee et al. demonstrated a 41-fold increase in the robotic treatment of colorectal cancer between 2004 and 2012 [26,27]. Halabi *et al* found a similar increase when looking at colorectal surgery in the United States and noted that while RACS is increasing in all hospital settings, it is being implemented at a greater rate in urban teaching centers [28]. The rise in the utilization of robotic surgery has been attributed to a myriad of factors. The appeal of the improved ergonomics seems to play a role but is difficult to validate [29]. Industry involvement with academic centers in the implementation of this device among training centers throughout the country, combined with the centralization of medical care, propels patients towards high volume large budget centers [26,[30], [31], [32]]. Patients themselves are contributing towards this trend as hospitals, and hospital systems, compete to maintain minimum procedure volumes. This process is occurring despite a lack of objective data on the quality differences in care [[33], [34], [35]]. Despite an overall shorter length of stay, robotic surgical patients had a significantly greater median hospital charge when compared to the laparoscopic or open approach. The similarity in cost between the groups is likely in part due to the shorter length of stay as well as the potential reduction in postoperative complications in the robotic group.

The overall reduction in postoperative morbidity and mortality in laparoscopic compared to open colorectal surgery in the elderly has been well documented [16,36,37]. In contrast, most studies comparing short-term outcomes between laparoscopic and robotic proctectomy have not shown a significant difference in complication rate between the two groups [[38], [39], [40], [41]]. While there appeared to be a potential trend towards decreased complications with the robotic group, we are unable to make a full assessment due to the sample size. As additional year groups are released by the NIS, further investigations may be performed using an adequately powered dataset to further elucidate these trends in improved outcomes with a robotic operation.

A significantly shorter LOS was seen in following a robotic operation compared to the laparoscopic and open procedures. Multiple meta-analyses comparing laparoscopic and open colorectal resection in the elderly have shown a reduced length of stay in patients undergoing a minimally invasive approach [37,42]. Conversely, previous studies have demonstrated an equivalent length of stay between a robotic and a laparoscopic resection for rectal cancers [28,[38], [39], [40], [41],^43^]. However, the mean age reported in these studies was 55–65 years old, a cohort significantly younger than those presented here. A recent study published in the Journal of Robotic Surgery shows similar operative times between laparoscopic and robotic approaches in colon and rectal resection. Furthermore, it has been shown in some series that operative and oncologic outcomes are similar between the two approaches in those 70 years of age and older. This was shown to be the case even with longer operative times in elderly patients compared with younger patients (300.6 min versus 214.5 min p = 0.03). The reason for the longer operative times was not fully explained although it does raise concern for the possibility of inappropriate matching in one particular study [44,45]. The aggregate of this data does point towards if not a benefit of robotic assisted surgery, then at least an equivalency in a myriad of age groups. Hence with the data shown here, the reduction in length of stay identified in the robotic group may act as a surrogate marker for an overall improvement in postoperative outcomes.

The discussion of costs and charges in regards to the robotic approach is not a new one. Other published data show the robotic approach total hospitalization cost to be 131% of the cost of the open approach. The data presented here shows the robotic approach to be 117% of the cost of an open approach hospitalization. As mentioned in the results section, the difference in charges appears to be more robust than the difference in cost [46]. It is not clear from this data why that is but it does warrant further monitoring to see if the difference diminishes with time.

We do acknowledge that there are inherent limitations in the utilization of the Nationwide Inpatient Sample. First, this is a retrospective study, and these results only demonstrate an association between findings. The NIS is an administrative database, and therefore has the potential for data entry error and missing data. Due to the design of this database, we are limited by the type and number of complications that could be evaluated, and there is no means of collecting long-term data following hospital discharge. Comorbidities and complications may also be under-coded when utilizing the *ICD-*9-CM system to collect data when compared to a clinical dataset such as NSQIP. Furthermore, there is an inherent selection bias among the pooled data from each institution, as well as variability among surgeon skill level and experience which cannot be accounted for in this database. In addition, implementation of robotic proctectomy in the elderly population is still in its infancy, as evidenced by only 75 reported cases in the databanks most recent year group (2013). This results in an underpowered sample size, limiting the potential data analysis and conclusions that could be ascertained. This study is unable to evaluate both the short and long-term oncologic outcomes, including resection margins and lymph node yield, findings which are critical to rectal cancer care. Despite these limitations, this study demonstrates the increasing adoption of robotic proctectomy for elderly individuals with similar and potentially improved postoperative outcomes when compared to a laparoscopic and an open approach.

## 5 Conclusion

Robotic proctectomy is growing in popularity. This, in concert with an ageing American population, necessitates further studies of the potential risks and benefits of this procedure. These results represent the largest analysis of robotic proctectomy in this population, and support the safety and efficacy of this approach in the elderly. This study provides a foundation for further investigations to fully elucidate the benefit of this operation in the treatment of rectal cancer.

## Ethical approval

The Tripler Army Medical Center IRB approved this study: TAMC 16N41.

## Sources of funding

There were no funding sources for this research.

## Author contribution

Carly Richards: study design, data analysis, writing.

Scott Steele: study design, data analysis.

Michael Lustik: data collection, data analysis.

Suzanne Gillern: data analysis, writing.

Justin Brady: data analysis, writing.

Ali Althans: data analysis, writing.

Robert Lim: data analysis, writing.

Andrew Schlussel: study design, data analysis, writing.

## Conflicts of interest

Dr. Richards, Dr. Steele, Mr. Lustik, Dr. Gillern, Dr. Brady, Ms. Althans, and Dr. Schlussel have no conflicts of interest or financial ties to disclose.

## Research registration number

NCT03765411.

## Guarantor

Carly Richards.

## Provenance and peer review

Not commissioned, externally peer reviewed.

## Disclosures

The authors of this paper have no conflicts of interest or financial ties disclose. The views expressed in this manuscript are those of the authors and do not reflect the official policy or position of our employer.

## References

[1] He W., Goodkind D., Kowal P. (2015). An aging world. International Population Reports.

[2] (2012 Oct). Guiding principles for the care of older adults with multimorbidity: an approach for clinicians. Guiding principles for the care of older adults with multimorbidity: an approach for clinicians: American Geriatrics Society Expert Panel on the Care of Older Adults with Multimorbidity. J. Am. Geriatr. Soc..

[3] Fielding L.P., Phillips R.K., Hittinger R. (1989 Mar 18). Factors influencing mortality after curative resection for large bowel cancer in elderly patients. Lancet.

[4] Tan K.Y., Kawamura Y., Mizokami K., Sasaki J., Tsujinaka S., Maeda T. (2009 Feb). Colorectal surgery in octogenarian patients--outcomes and predictors of morbidity. Int. J. Colorectal Dis..

[5] Law W.L., Chu K.W., Tung P.H. (2002 Dec). Laparoscopic colorectal resection: a safe option for elderly patients. J. Am. Coll. Surg..

[6] Cheung H.Y., Chung C.C., Fung J.T., Wong J.C., Yau K.K., Li M.K. (2007 Nov). Laparoscopic resection for colorectal cancer in octogenarians: results in a decade. Dis. Colon Rectum.

[7] Stocchi L., Nelson H., Young-Fadok T.M., Larson D.R., Ilstrup D.M. (2000 Mar). Safety and advantages of laparoscopic vs. open colectomy in the elderly: matched-control study. Dis. Colon Rectum.

[8] Vignali A., Di Palo S., Tamburini A., Radaelli G., Orsenigo E., Staudacher C. (2005 Nov). Laparoscopic vs. open colectomies in octogenarians: a case-matched control study. Dis. Colon Rectum.

[9] Champagne B.J., Delaney C.P. (2007 Aug). Laparoscopic approaches to rectal cancer. Clin. Colon Rectal Surg..

[10] Jayne D.G., Brown J.M., Thorpe H., Walker J., Quirke P., Guillou P.J. (2005 Sep). Bladder and sexual function following resection for rectal cancer in a randomized clinical trial of laparoscopic versus open technique. Br. J. Surg..

[11] van der Pas M.H., Haglind E., Cuesta M.A., Furst A., Lacy A.M., Hop W.C. (2013 Mar). Laparoscopic versus open surgery for rectal cancer (COLOR II): short-term outcomes of a randomised, phase 3 trial. Lancet Oncol..

[12] Park S., Kim N.K. (2015 Jul). The role of robotic surgery for rectal cancer: overcoming technical challenges in laparoscopic surgery by advanced techniques. J. Korean Med. Sci..

[13] Baik S.H., Kwon H.Y., Kim J.S., Hur H., Sohn S.K., Cho C.H. (2009 Jun). Robotic versus laparoscopic low anterior resection of rectal cancer: short-term outcome of a prospective comparative study. Ann. Surg. Oncol..

[14] Kim J.Y., Kim N.K., Lee K.Y., Hur H., Min B.S., Kim J.H. (2012 Aug). A comparative study of voiding and sexual function after total mesorectal excision with autonomic nerve preservation for rectal cancer: laparoscopic versus robotic surgery. Ann. Surg. Oncol..

[15] D'Annibale A., Pernazza G., Monsellato I., Pende V., Lucandri G., Mazzocchi P. (2013 Jun). Total mesorectal excision: a comparison of oncological and functional outcomes between robotic and laparoscopic surgery for rectal cancer. Surg. Endosc..

[16] Frasson M., Braga M., Vignali A., Zuliani W., Di Carlo V. (2008 Mar). Benefits of laparoscopic colorectal resection are more pronounced in elderly patients. Dis. Colon Rectum.

[17] Jayne D., Pigazzi A., Marshall H., Croft J., Corrigan N., Copeland J. (2017 Oct 24). Effect of robotic-assisted vs conventional laparoscopic surgery on risk of conversion to open laparotomy among patients undergoing resection for rectal cancer: the ROLARR randomized clinical trial. J. Am. Med. Assoc..

[18] Agha R.A., Borrelli M.R., Vella-Baldacchino M., Thavayogan R., Orgill D.P., STROCSS Group (2017 Oct). The STROCSS statement: strengthening the reporting of cohort studies in surgery. Int. J. Surg..

[19] (2017). Overview of the Nationwide Inpatient Sample.

[20] Charlson M.E., Pompei P., Ales K.L., MacKenzie C.R. (1987). A new method of classifying prognostic comorbidity in longitudinal studies: development and validation. J. Chronic Dis..

[21] Elixhauser Anne, Claudia Steiner, Harris Robert, Coffey Rosanna (1998). Comorbidity measures for use with administrative data. Med. Care.

[22] (2014). All Patient Refined Diagnosis Related Groups (APR-DRGs), Version 20.0. Methodology Overview.

[23] Washington C.W., Derdeyn C.P., Dacey R.G., Dhar R., Zipfel G.J. (2014 Aug). Analysis of subarachnoid hemorrhage using the nationwide inpatient sample: the NIS-SAH severity score and outcome measure. J. Neurosurg..

[24] Singh J.A., Kwoh C.K., Boudreau R.M., Lee G.C., Ibrahim S.A. (2011 Aug). Hospital volume and surgical outcomes after elective hip/knee arthroplasty: a risk-adjusted analysis of a large regional database. Arthritis Rheum..

[25] Schlussel A.T., Lustik M.B., Johnson E.K., Maykel J.A., Champagne B.J., Damle A. (2016 Mar). A nationwide assessment comparing nonelective open with minimally invasive complex colorectal procedures. Colorectal Dis..

[26] Damle A., Damle R.N., Flahive J.M., Schlussel A.T., Davids J.S., Sturrock P.R. (2017 Mar 21, Page 820-824). Diffusion of technology: trends in robotic-assisted colorectal surgery. Am. J. Surg..

[27] Lee M.G., Chiu C.C., Wang C.C., Chang C.N., Lee S.H., Lee M. (2017 May 17). Trends and outcomes of surgical treatment for colorectal cancer between 2004 and 2012- an analysis using national inpatient database. Sci. Rep..

[28] Halabi W.J., Kang C.Y., Jafari M.D., Nguyen V.Q., Carmichael J.C., Mills S. (2013 Dec). Robotic-assisted colorectal surgery in the United States: a nationwide analysis of trends and outcomes. World J. Surg..

[29] Goldstraw M.A., Challacombe B.J., Patil K., Amoroso P., Dasgupta P., Kirby R.S. (2012 Mar). Overcoming the challenges of robot-assisted radical prostatectomy. Prostate Cancer Prostatic Dis..

[30] Riikonen J., Kaipia A., Petas A., Horte A., Koskimaki J., Kahkonen E. (2016 Jun). Initiation of robot-assisted radical prostatectomies in Finland: impact on centralization and quality of care. Scand J Urol.

[31] Sammon J.D., Karakiewicz P.I., Sun M., Sukumar S., Ravi P., Ghani K.R. (2013 Apr). Robot-assisted versus open radical prostatectomy: the differential effect of regionalization, procedure volume and operative approach. J. Urol..

[32] Yu H.Y., Hevelone N.D., Lipsitz S.R., Kowalczyk K.J., Nguyen P.L., Hu J.C. (2012 May). Hospital volume, utilization, costs and outcomes of robot-assisted laparoscopic radical prostatectomy. J. Urol..

[33] Aggarwal A., Lewis D., Mason M., Purushotham A., Sullivan R., van der Meulen J. (2017 Nov). Effect of patient choice and hospital competition on service configuration and technology adoption within cancer surgery: a national, population-based study. Lancet Oncol..

[34] Sugihara T., Yasunaga H., Matsui H., Nagao G., Ishikawa A., Fujimura T. (2017 Jul 1). Accessibility to surgical robot technology and prostate-cancer patient behavior for prostatectomy. Jpn. J. Clin. Oncol..

[35] Aggarwal A., Lewis D., Charman S.C., Mason M., Clarke N., Sullivan R. (2018 Jun). Determinants of patient mobility for prostate cancer surgery: a population-based study of choice and competition. Eur. Urol..

[36] Antoniou S.A., Antoniou G.A., Koch O.O., Pointner R., Granderath F.A. (2015 Feb). Laparoscopic colorectal surgery confers lower mortality in the elderly: a systematic review and meta-analysis of 66,483 patients. Surg. Endosc..

[37] Seishima R., Okabayashi K., Hasegawa H., Tsuruta M., Shigeta K., Matsui S. (2015 Apr). Is laparoscopic colorectal surgery beneficial for elderly patients? A systematic review and meta-analysis. J. Gastrointest. Surg..

[38] Tam M.S., Kaoutzanis C., Mullard A.J., Regenbogen S.E., Franz M.G., Hendren S. (2016 Feb). A population-based study comparing laparoscopic and robotic outcomes in colorectal surgery. Surg. Endosc..

[39] Baek J.H., Pastor C., Pigazzi A. (2011 Feb). Robotic and laparoscopic total mesorectal excision for rectal cancer: a case-matched study. Surg. Endosc..

[40] Park J.S., Choi G.S., Lim K.H., Jang Y.S., Jun S.H. (2010 Dec). Robotic-assisted versus laparoscopic surgery for low rectal cancer: case-matched analysis of short-term outcomes. Ann. Surg. Oncol..

[41] Keller D.S., Senagore A.J., Lawrence J.K., Champagne B.J., Delaney C.P. (2014 Jan). Comparative effectiveness of laparoscopic versus robot-assisted colorectal resection. Surg. Endosc..

[42] Li Y., Wang S., Gao S., Yang C., Yang W., Guo S. (2016 Mar). Laparoscopic colorectal resection versus open colorectal resection in octogenarians: a systematic review and meta-analysis of safety and efficacy. Tech. Coloproctol..

[43] Park J.S., Choi G.S., Lim K.H., Jang Y.S., Jun S.H. (2011 Jan). S052: a comparison of robot-assisted, laparoscopic, and open surgery in the treatment of rectal cancer. Surg. Endosc..

[44] Nolan H.R., Smith B.E., Honaker M.D. (2018 Dec). Operative time and length of stay is similar between robotic assisted and laparoscopic colon and rectal resections. J Robot Surg.

[45] de'Angelis N., Abdalla S., Bianchi G., Memeo R., Charpy C., Petrucciani N. (2018 Nov). Robotic versus laparoscopic colorectal cancer surgery in elderly patients: a propensity score match analysis. J. Laparoendosc. Adv. Surg. Tech..

[46] Silva-Velazco J., Dietz D.W., Stocchi L., Costedio M., Gorgun E., Kalady M.F. (2017 May). Considering value in rectal cancer surgery: an analysis of costs and outcomes based on the open, laparoscopic, and robotic approach for proctectomy. Ann. Surg..

